# Real-World Evidence of Transcutaneous Afferent Patterned Stimulation for Essential Tremor

**DOI:** 10.5334/tohm.715

**Published:** 2022-09-01

**Authors:** Salima Brillman, Kalea Colletta, Sally Borucki, Peter T. Lin, Olga Waln, Melita Petrossian, Pravin Khemani, Apoorva Rajagopal, Kathryn H. Rosenbluth, Dhira Khosla

**Affiliations:** 1 Parkinson’s Disease and Movement Disorders Center of Silicon Valley, Palo Alto, CA, USA; 2 Department of Neurology, Edward Hines Jr. VA Hospital, Hines, IL, USA; 3 Department of Neurology, Temple VA Medical Center, Temple, TX, USA; 4 Valley Parkinson Clinic, Los Gatos, CA, USA; 5 Houston Methodist Neurological Institute, Houston, TX, USA; 6 Pacific Neuroscience Institute, Pacific Movement Disorders Center, Santa Monica, CA, USA; 7 Swedish Neuroscience Institute, Seattle, WA, USA; 8 Cala Health Inc, San Mateo, CA, USA

**Keywords:** peripheral neuromodulation, essential tremor, real-world evidence, remote sensing, transcutaneous afferent patterned stimulation

## Abstract

**Background:**

Transcutaneous afferent patterned stimulation (TAPS) is a prescription, wrist-worn device-delivered, non-invasive neuromodulation therapy for treatment of hand tremor in patients with essential tremor (ET). This retrospective post-market surveillance study evaluated real-world effectiveness of TAPS from patients using therapy on-demand for at least 90 days between August 2019 through June 2021.

**Methods:**

Demographics were summarized from TAPS prescriptions received from the patient’s healthcare provider. Therapy usage and effectiveness were analyzed from device logs, which included tremor measurements from onboard motion sensors. Tremor history and patient-reported outcomes were assessed from a voluntary survey.

**Results:**

A total of 321 patients (average age 71 years, 32% female) met the criteria for this analysis, 216 of whom had tremor measurements available for analysis and 69 of whom completed the survey. Total usage period ranged from 90 to 663 days, with 28% of patients using the device for over one year. Patients used therapy 5.4 ± 4.5 (mean ± 1 standard deviation) times per week. TAPS reduced tremor power by 71% (geometric mean) across all sessions, with 59% of patients experiencing >50% tremor reduction after their sessions. Eighty-four percent (84%) of patients who returned the voluntary survey reported improvement in at least one of eating, drinking, or writing, and 65% of patients reported improvement in quality of life. Self-reported device-related safety complaints were consistent with adverse events in prior clinical trials.

**Discussion:**

Real-world evidence is consistent with prior clinical trials and confirms TAPS provides safe and effective tremor control for many patients with ET. Future work assessing multi-year safety and effectiveness would be valuable to extend these data.

## Introduction

Essential tremor (ET) is one of the most common movement disorders in adults. It affects approximately seven million adults in the United States and disproportionately impacts older individuals [1,2]. ET symptoms, characterized mostly by upper limb tremor, often impair ability to independently carry out activities of daily living, negatively impact mental health, and reduce quality of life for those afflicted [3,4,5].

Treatment options for ET are limited in efficacy and accessibility. Pharmacotherapy to treat ET symptoms typically include non-selective beta blockers (e.g., propranolol), anticonvulsants (e.g., primidone, topiramate, gabapentin), and benzodiazepines (e.g., clonazepam) [6,7,8,9]. While these medications can successfully control tremor for some patients, many patients opt to forego or discontinue pharmacotherapy due to lack of effectiveness or intolerable side-effects at doses required to treat tremor [10]. Botulinum toxin injections and neurosurgical procedures (e.g., focused ultrasound, thalamotomy, deep brain stimulation) are alternative treatment options for patients who fail pharmacotherapy, but these treatments are likewise inaccessible to many patients due to cost and associated risk [11,12,13]. Patients with ET who do not respond to or do not prefer these typical treatments are left with limited options to manage their tremor burden.

Transcutaneous afferent patterned stimulation (TAPS) is an on-demand, non-invasive, peripheral neuromodulation therapy cleared by the United States Food and Drug Administration (FDA) in 2018 for treatment of ET hand tremor symptoms [14]. Recent guidance has incorporated TAPS therapy into ET treatment guidelines for healthcare providers (HCPs) as an adjunctive first-line treatment (with pharmacotherapy) and prior to or in combination with second and third-line treatments [9]. Patients using TAPS therapy are instructed to self-administer stimulation as-needed to control their tremor, with prior studies indicating 60 minutes of tremor relief following a 40-minute therapy session for many individuals [15,16]. TAPS therapy consists of stimulation of the median and radial nerves at the wrist at a frequency individualized to each patient’s tremor [17]. TAPS is hypothesized to control ET symptoms by dephasing neural oscillations at the Ventral Intermediate (VIM) nucleus, the thalamic relay where deep brain stimulation (DBS) is implanted for the treatment of ET [18,19,20,21]. In a randomized single-session clinical trial, the magnitude of improvement across tasks in the treatment group corresponded to a 49% reduction in tremor according to patient’s self-rated quality of life as measured by Bain and Findley Activities of Daily Living (BF-ADL) and a 42% reduction according to physician-rated Tremor Research Group Essential Tremor Rating Scale (TETRAS) [17].

In a 3-month prospective clinical trial, the proportion of patients rated “Severe” or “Moderate” improved from 49.3% (TETRAS) and 64.8% (BF-ADL) at baseline to 21.0% (TETRAS) and 23.0% (BF-ADL) at study exit [15].

Closely monitored clinical trials are the gold standard to evaluate a treatment’s safety and efficacy, but findings from clinical trials do not always translate to a real-world environment where patients may not receive the same level of clinical supervision and instruction. This retrospective analysis aimed to build on previous prospective clinical studies by assessing real-world usage, effectiveness, and safety of TAPS therapy. This study leveraged the cloud-connected nature of the TAPS device which allowed for analysis of data collected in an unsupervised commercial home-use setting.

## Methods

### Device and data

**Figure 1:**
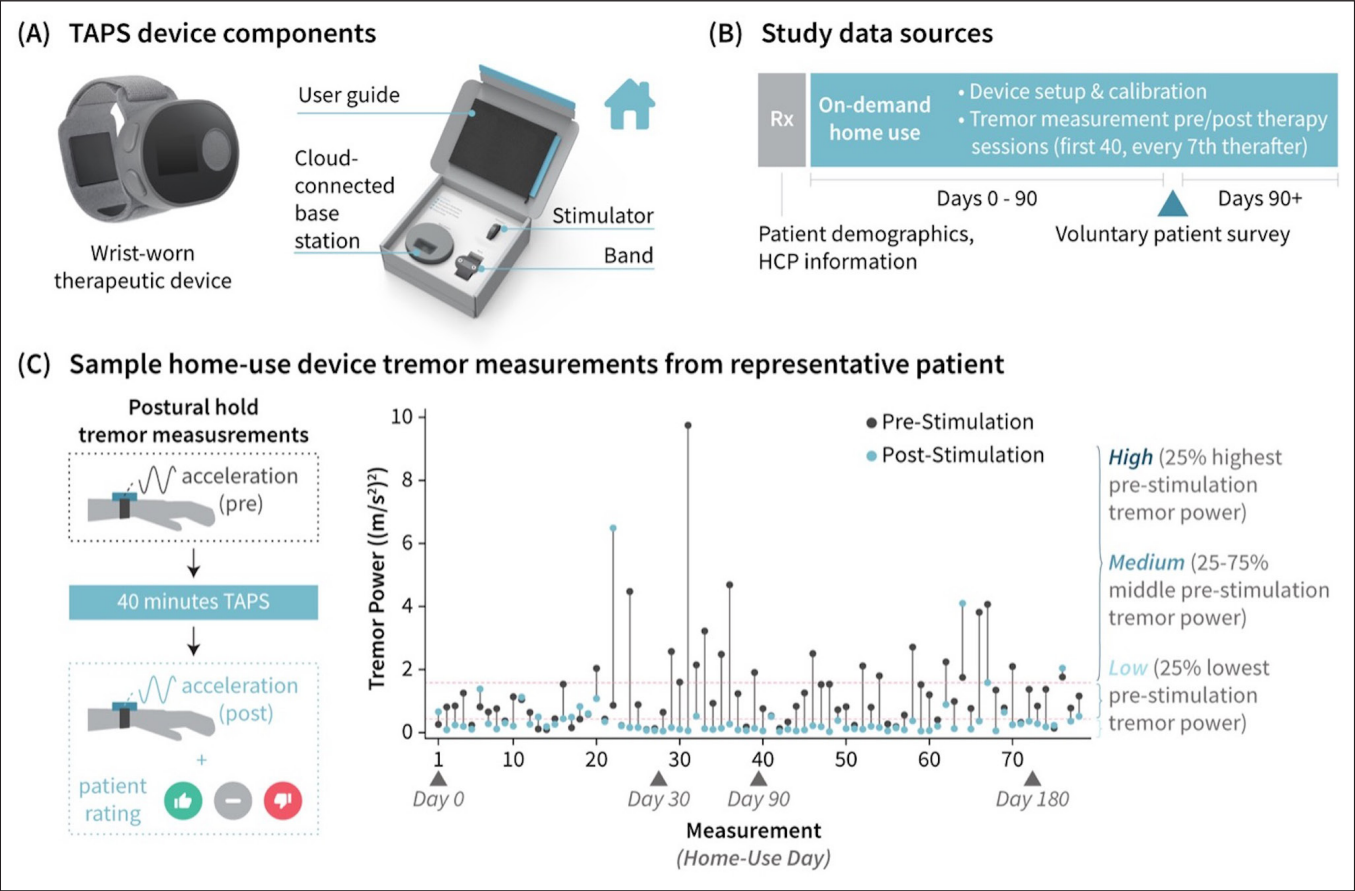
**TAPS device and data. (A)** The prescription wrist-worn TAPS device was shipped directly to patients and was comprised of a stimulator, band containing TAPS-delivering electrodes, and cloud-connected base station that charged the device and streamed device logs to a centralized database. A user guide included with the device contained instructions for patients on setup, calibration, and usage of TAPS therapy. **(B)** Study data were derived from the healthcare provider-completed prescription form, device logs automatically generated during therapy home use, and a voluntary survey sent to patients after 90 days of therapy use. **(C)** For the first 40 sessions and every 7^th^ session thereafter, the device prompted patients to perform a twenty-second postural hold before and after stimulation and prompted patients to self-rate their tremor improvement after stimulation. Tremor power, computed from postural hold accelerometry data of clean signal quality, was used to characterize tremor severity. Additionally, measurements were classified into patient-specific bands of High, Medium, and Low based on pre-stimulation tremor power.

Real-world performance of TAPS therapy, delivered with a wrist-worn device (Cala Trio™, Cala Health, San Mateo, CA, USA), was analyzed for patients who had a diagnosis of ET reported by their prescribing HCP, had used TAPS therapy for at least 90 days, and had a minimum of 10 sessions in device logs. The TAPS device consisted of a (1) wrist-worn stimulator with a triaxial accelerometer that generated the TAPS waveform and logged all device-collected data, (2) detachable wrist band with electrodes configured to target the median and radial nerves, and (3) cloud-connected base station that charged the stimulator and securely transmitted all device data to a centralized database (Figure 1A). The instructions for use provided with the device instructed patients on how to calibrate and use TAPS therapy.

Demographic information and tremor history were gathered from the HCP prescription form and from a voluntary survey (eTable 1, in Supplemental materials) sent to patients after 90 days of therapy use (Figure 1B). Usage and effectiveness information were compiled from device logs, which included (1) timestamps of all sessions; and (2) device-prompted postural hold tremor accelerometry measurements and (3) patients’ self-rating of post-session impression of tremor (improved, no change, worsened) collected before and after the first forty therapy sessions and every seventh session thereafter (Figure 1B).

**eTable 1:** Patient voluntary survey.


SURVEY QUESTION

For how many years have you had hand tremor symptoms? *[select one]* ◦ Less than 5 years ◦ 5 – 10 years ◦ 10 – 20 years ◦ Greater than 20 years

How would you rate the overall tremor severity of your treated hand without using Cala Trio? *[select one]* ◦ Mild – mild tremor not causing difficulty in performing any activities ◦ Moderate – tremor causes difficulty in performing some activities ◦ Marked – tremor causes difficulty in performing most or all activities ◦ Severe – tremor prevents performing some activities

How many tremor medications have you tried prior to starting Cala Trio? *[select one]* ◦ None ◦ 1 ◦ 2 ◦ 3 ◦ 4 or more

How many tremor medications are you currently taking for your hand tremor? *[select one]* ◦ None ◦ 1 ◦ 2 ◦ 3 ◦ 4 or more

Have you changed your tremor medication dosage since starting Cala Trio? *[select one]* ◦ Discontinued use ◦ Reduced dosage ◦ Increased dosage ◦ Not changed, but plan to consult my doctor ◦ Not changed, nor do I plan to ◦ I am not on tremor medications

If Cala Trio were presented as an option at the same time as medications or surgical procedures, which would you choose? (1 = would choose first) *[rank preferences from 1 to 3]* • Cala Trio • Tremor Medications • Surgical procedures

Which activities would you most like Cala Trio therapy to help you with? (1 = Most important to you) *[rank importance from 1 to 5]* • Activities of daily living (e.g., eating, drinking) • Social activities • Hobbies • Professional responsibilities/work • Housework

Please rate the impact the Cala Trio has on the following activities. *[for each, select “Much Improved”, “Improved”, “No Change”, “Worsened”, or “Much Worsened”]* • Eating • Drinking • Handwriting • Social activities (e.g., dining with friends) • Medication management (e.g., opening pill bottle, eye drops, checking blood sugar) • Professional responsibilities/work (e.g., computer, phone, meetings, presentations) • Housework (e.g., cooking, fixing small things) • Personal hygiene/getting dressed (e.g., hair, makeup, shaving, tying a tie, buttons) • Hobbies (e.g., music, knitting, fishing, art) • Overall quality of life


### Usage and effectiveness analysis

Usage was summarized as an average number of completed sessions (of at least 20 minutes in duration) per week.

Effectiveness was primarily assessed by measuring improvements in tremor power (computed from device postural hold accelerometry data) from before to after a therapy session. Tremor power is a metric capturing the frequency and amplitude of tremor motion and has been previously correlated to clinical gold-standard tremor assessments [15,16,22,23]. The spectral peak was identified statistically within the 4–12 Hz range expected for essential tremor, after which tremor power was integrated over the 3 Hz window centered on the identified spectral peak. Each patient’s effectiveness was reported as the median improvement ratio across all sessions, where improvement ratio was defined as the ratio between tremor power before each session to the tremor power after each session and estimated improvements in tremor amplitude. Detailed descriptions of tremor power and improvement ratio have been previously published [15]. Consistent with prior clinical trials, sessions with incomplete or motion artifact-contaminated tremor measurements were excluded due to poor data quality, and sessions started within 120 minutes of a prior session were excluded to avoid carry-over treatment effect in effectiveness analysis [15,16]. Patients with at least 10 sessions following these exclusions were included in the effectiveness analysis. Improvement ratios were tested against a null hypothesis of no improvement using a Wilcoxon sign-rank test.

Additionally, each session by each patient was classified according to whether the patient’s tremor was “High”, which was defined as tremor in that patient’s highest quartile, “Medium”, defined as the middle quartiles, or “Low”, defined as the lowest quartile. (Figure 1C). Median improvement ratios and post-stimulation tremor classification was then calculated within each classification group (i.e., each patient’s “High”, “Medium” and “Low”).

Finally, to build upon evidence from a previous clinical study that found no evidence of habituation to TAPS therapy (i.e., loss of effect due to developed tolerance to stimulation) after 90 days of repeated use [15], patients in this real-world study who had used therapy for at least 1 year were assessed for habituation by comparing their TAPS effectiveness in their first 90 days of use to effectiveness after 90 days. Effectiveness in each period (before and after 90 days) was characterized as median tremor power improvement in each period.

The frequencies at which patients rated a session as “Improved”, “No change”, or “Worsened” were averaged across the same sessions used to assess improvements in tremor power. Patients’ self-reports on effect of and preference for TAPS therapy were summarized descriptively from the voluntary patient survey. Survey questions were designed by the device manufacturer (Cala Health) and based on existing validated patient assessments where appropriate.

Usage and effectiveness data were also descriptively summarized for the subgroup of patients <65 and ≥65 years of age.

### Complaints Analysis

The on-market complaint database from the 321 patients in this retrospective study were evaluated for the type and frequency of adverse events reported as complaints. Only adverse events reported to the manufacturer were available for analysis, while adverse events not reported to the manufacturer, such as adverse events patients shared only with their physician, were not available for analysis.

## Results

The study included 321 patients with ET who met the 90-day, 10-session inclusion criteria. Total use period ranged from 90 to 663 days, with 28% of patients (89 of 321) having used therapy for greater than one year. These 321 patients performed 70,635 therapy sessions that were included in the usage analysis; and 216 of the 321 patients performed 9,163 therapy sessions that met the analysis criteria for effectiveness (i.e. having performed at least 10 sessions that included postural holds, were at least 20 minutes in duration, and were completed at least 120 minutes after the previous session). Sixty-six percent of stimulation sessions analyzed for efficacy had high quality and artifact-free postural hold data from both before and after stimulation for inclusion in the improvement ratio analysis. As this was a retrospective analysis of real-world evidence, the postural holds before or after the stimulation session were not performed in a controlled environment, and therefore it is difficult to assess what types of motion contaminated the signal during at-home unsupervised usage.

Patients’ average age was 71 (13) years (standard deviation, SD) and was 32% female (N = 321, Table 1). TAPS therapy prescribers were primarily neurologists (71%), including movement disorder specialists (26%), but also included other specialties. Of the patients returning the voluntary survey (N = 69), 62% reported having 10 or more years of hand tremor symptoms, 96% self-rated their tremor severity as moderate or greater, 88% reported having tried at least one medication to manage their tremor prior to trying TAPS therapy, and 78% rated activities of daily living (ADLs) as the most important area of therapeutic need (Table 1). Patients returning the voluntary survey had an average age of 68 (11) years and were 38% female. Patients used therapy on-demand an average of 5.4 (4.5) times per week over their total use period (Table 2; N = 321), which included an average 4.6 completed sessions per week over days 180 – 360 (N = 89) and 4.0 sessions per week beyond 360 days (N = 89).

**Table 1:** Study population.


POPULATION CHARACTERISTICS*	

**Age (years, mean ± SD)**	71 ± 10

**Gender (% female)^+^**	32%

**TAPS prescriber specialty**	

Neurologists (movement disorder specialists)	26%

Neurologists (general, and other sub-specialists)	45%

Family practice, internal medicine	14%

Occupational or physical therapist	3%

Other (incl. unknown)	12%

Patient-reported tremor burden**	

**Years with tremor symptoms**	

<5 years	13%

5–10 years	25%

10–20 years	30%

>20 years	32%

**Self-rated pre-TAPS tremor severity**	

Mild	4%

Moderate	62%

Marked	25%

Severe	9%

**Number medications tried prior to TAPS**	

None	12%

1	22%

2	25%

3	19%

>4	23%

**Number of current medications for tremor**	

None	38%

1	41%

2	17%

3	4%

>4	0%

**Most important area of therapeutic need**	

Activities of daily living	78%

Social activities	6%

Hobbies	7%

Professional responsibilities	9%

Housework	0%


* From N = 321 (full study population) prescription forms. ^+^ *From N = 121 of the 321 for whom gender data was available*. ** From N = 69 survey respondents.

**Table 2:** Descriptive Statistics of Usage and Effectiveness.


	ALL PATIENTS	AGE <65	AGE ≥ 65

**Usage patterns**, mean (SD)			

Sessions per week	5.4 (4.5)	4.8 (5.8)	5.5 (4.2)

Days per week with at least one session	3.2 (1.9)	2.6 (2.0)	3.4 (1.9)

Sessions per day on days when therapy used	1.5 (0.6)	1.5 (0.7)	1.5 (0.6)

**Device-measured outcomes**, geometric mean (×geometric SD)*			

Improvement ratio, all sessions	3.5 (×4.1)	4.4 (×3.2)	3.3 (×4.3)

Improvement ratio, “High” tremor sessions	9.1 (×6.2)	15.9 (×7.2)	8.1 (×5.9)

Improvement ratio, “Medium” tremor sessions	3.7 (×4.6)	4.6 (×3.4)	3.5 (×4.9)

Improvement ratio, “Low” tremor sessions	1.3 (×3.3)	1.2 (×2.2)	1.4 (×3.6)

**Patient-rated outcomes**			

% Sessions rated “Improved”	59%	69%	57%

% Sessions rated “No Change”	38%	29%	40%

% Sessions rated “Worsened”	3%	2%	3%


* Geometric mean and SD are analogous to arithmetic mean and SD of log-transformed data; and geometric SD represents ×/÷ factor change from geometric mean.

**Figure 2:**
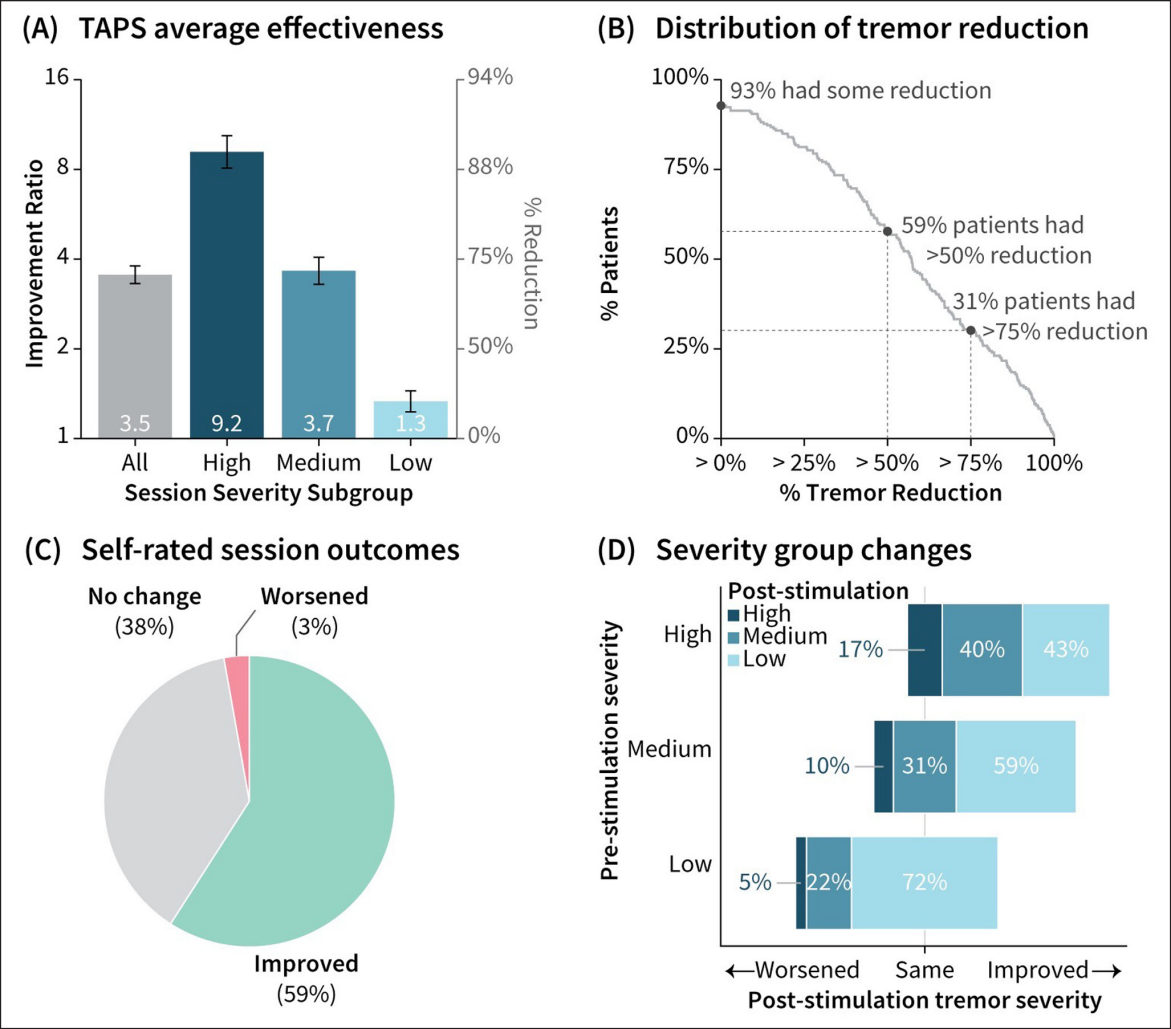
**Effectiveness assessed through longitudinal home-use data. (A)** TAPS effectiveness was summarized across all, and High/Medium/Low session groups. Error bars represent geometric mean ×/÷ 1 geometric standard error (equivalent in range to mean ± 1 standard error of log-transformed data). **(B)** Across all sessions, cumulative distribution of per-patient tremor reductions indicated clinically meaningful improvement for most patients. **(C)** Patient self-ratings of post-TAPS improvement were similar in distribution to motion-sensor ratings of post-TAPS improvement. **(D)** Frequency of post-stimulation tremor severity categories were broken down for each pre-stimulation severity group. TAPS improved tremor severity category for most sessions that started High or Medium, and maintained Low tremor for sessions that started Low.

Tremor power decreased by 3.5 (×4.1) fold (i.e., 71% reduction; geometric mean (×geometric SD); p ≪ 0.001; Figure 2A), with 59% of patients experiencing at least a 2-fold (i.e., 50%) reduction (Figure 2B). Patients completing multiple qualifying sessions in a single day (N = 186 of 216) experienced similar tremor reduction from their second session of the day as from their first session of the day (improvement ratio 3.7 vs 3.5, respectively). Patient ratings on the device (Figure 2C) were consistent with these device-measured tremor improvements (ρ = 0.45, Spearman’s correlation coefficient).

Analysis by quartiles of tremor severity showed patients experienced the greatest tremor reductions when they used TAPS when their tremors were most severe (Figure 2A; Table 2). At times of “High” pre-stimulation tremor, patients experienced a 9.1 (×6.2) fold (i.e., 89%) tremor reduction after TAPS, with “High” tremor improving to “Medium” or “Low” ranges in 83% of sessions, on average (Figure 2D). At times of “Low” pre-stimulation tremor, TAPS maintained tremor in the “Low” range in 72% of sessions.

Analysis of the 87 back-to-back instances (from 40 patients) that had complete motion data found that these patients experienced a 2.4-fold improvement (geometric mean) in tremor power (p = 0.00013) after their first session (similar to single-session improvement in these patients’ other non-back-to-back sessions, p = 0.71) that was maintained or enhanced throughout their next session (4.4-fold improvement relative to the power prior to their first session; p = 0.000019).

For the patients who used therapy for over one year and met the criteria for the effectiveness analysis (N = 74 of 89), no significant habituation was observed; improvement ratios for these patients in the first 90 days were similar to those beyond 90 days (4.2 (×4.9) vs 5.2 (×7.1), respectively; p = 0.16).

**Figure 3:**
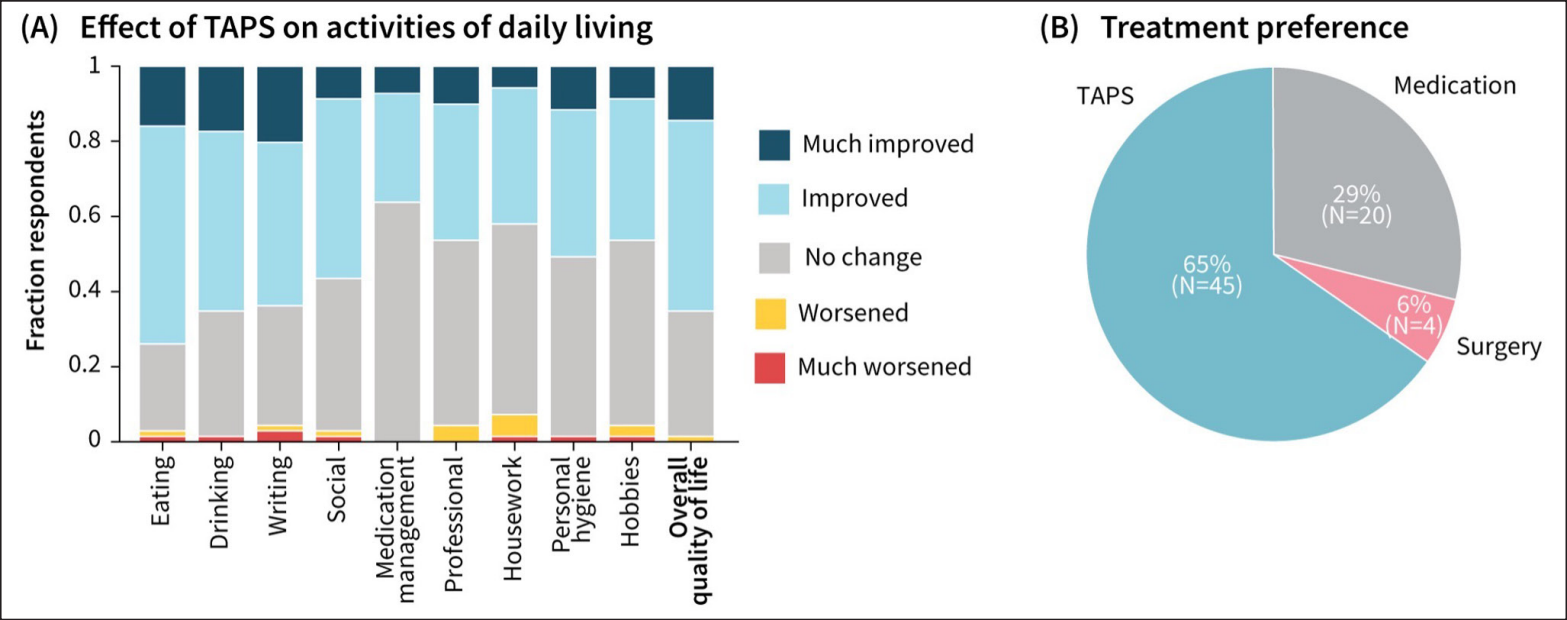
**Patient-reported outcomes. (A)** Respondents rated effect of TAPS on various activities of daily living, with 84% of respondents indicating improvement in at least one of eating, drinking, or writing, and 65% of respondents indicating improvements in overall quality of life. **(B)** Survey respondents generally preferred TAPS over medication or surgical management of tremor.

In the voluntary survey, patients indicated improvement in key activities of daily living (74% in eating, 65% in drinking, and 64% in writing), and 65% of patients indicated improvement in overall quality of life (Figure 3A). Two-thirds (65%) of patients indicated a preference for TAPS therapy over both medication and surgical intervention for tremor management; 29% (20 of 69) preferred medication for tremor management, with 18 of these 20 patients indicating TAPS was their second choice; and only 6% (4 of 69) preferred surgery (Figure 3B). Patients who ranked TAPS therapy as their preferred option over medication and surgery may directionally have greater TAPS effectiveness than those who did not (median improvement ratio 3.7 vs 1.8, respectively; p = 0.11). Of the patients who were on tremor medication before trying TAPS (43 of 69, or 62% of respondents), 24% reduced their tremor medication (14% discontinued) after 90 days, 4% increased their tremor medication, and the remaining 72% did not change (16% were considering a change, pending discussion with their HCP).

Device-related safety complaints from the 321 patients were consistent with adverse events reported in prior clinical trials (15). Overall, 12% of patients reported at least one safety-related complaint, including discomfort (e.g., electric shock, burning, pain, tingling, or numbness sensation; 5.4% patients) or skin irritation (itchiness, redness, or rash; 5.1% patients) at or near the stimulation site and physical symptoms (e.g., discomfort, pain, or stiffness outside of the stimulation site, headache, altered vision; 2.2% patients). No severe safety events were reported.

## Discussion

This retrospective real-world analysis of on-demand TAPS extended the therapy efficacy data established in a prior prospective clinical study in which patients were instructed to use therapy twice daily [15]. The real-world evidence demonstrated that patients continued to use therapy weekly over time and had effectiveness similar to prospective clinical trial findings (e.g., ≥2-fold tremor reduction for 59% of patients (Figure 2) compared to 54% of patients in a prior three-month study) [15]. Furthermore, this study’s results extend prior three-month efficacy data to suggest habituation or loss of effect was not observed in the 89 patients who used therapy for one year or more. These objective data coupled with self-reported quality of life improvement and patients’ preference for TAPS compared to existing standard-of-care options (Figure 3) reinforce that TAPS can be a valuable treatment option for patients with ET.

The unique ability to assess therapy usage and effectiveness directly on the therapeutic device enabled remote longitudinal monitoring with minimal patient burden. This and similar capabilities in other emerging connected devices provide tools for real world assessment of therapeutic effectiveness and patient behavior [24,25]. For example, the repeated at-home tremor measurements (Figure 1C) allowed broader characterization of the range of each patient’s tremor than is possible with typical single-session clinical studies. In this study, patients’ pre-stimulation postural hold tremor powers corresponded to TETRAS (Tremor Research Group ET Rating Assessment Scale [26]) scores of 2.0 (0.2; mean, SD), estimated using a previously published regression model [15]. However, the daily lows and highs for patients ranged from estimated TETRAS scores of 1.0 – 3.5; and for an individual patient, “High” and “Low” tremor instances differed by an average of 0.8 TETRAS points (range 0 – 2). Understanding this day-to-day variability allowed for characterization of therapy effectiveness across different needs (e.g., relief from “High” tremor versus prophylactic control during “Low” tremor). Passively monitored motion data would be valuable to further contextualize how these therapy-specific tremor measurements relate to tremor burden throughout the day, and therefore lead to better understanding of patients’ choice of when to use therapy.

Remote monitoring also facilitates discovery of new therapy use patterns. For example, exploratory post-hoc analysis identified 2,189 instances (from 143 patients, 46% of study population) where patients opted to complete two back-to-back 40-minute TAPS therapy sessions separated by less than 10 minutes. The first of these back-to-back sessions was included in the study’s effectiveness analyses, but the second was excluded to avoid influence of carry-over therapeutic effect in single-session effectiveness characterization. Overall rate of safety events in these patients was similar to the rate in patients not performing back-to-back sessions (15% vs 9%, respectively; p = 0.11). While patients’ intent in performing a back-to-back session cannot be known, the data suggest patients may have performed an additional session to extend the duration of therapeutic benefit beyond that achieved from a single session. Future studies could use these same device-integrated monitoring to explore the therapeutic benefits of modifications to TAPS dosing or waveform, and to inform optimal patient selection.

Previous TAPS studies reported an improvement in baseline tremor [15] and change in neural circuitry [18] with three months of twice-daily use; accordingly we evaluated this real-world evidence to see if similar trends could be seen, despite the dosing in this study being substantially lower (i.e., 5.4-sessions per week). Of the long-term (1+ year) patient cohort, 21% of patients had a ≥2-fold improvement (50% reduction) in their baseline tremor after 90 days, though there was no population-level statistical difference between baseline tremor power in days 0 – 90 compared to beyond 90. Further studies should explore other therapy paradigms to further improve baseline tremor, which may be particularly meaningful in a degenerative condition. Future larger studies should also include sensitivity and trending analyses broken out by months of usage, frequency of usage, baseline tremor characteristics and other variables.

Potential confounders arising in retrospective, observational, real-world, post-market studies should be noted while interpreting this study’s results. First, the inclusion criteria for the study’s device and patient self-reported usage and effectiveness analyses could have introduced bias. The 90-day inclusion criterion was chosen to allow patients sufficient time to resolve therapy use patterns and to mirror length of a prior clinical trial [15], but could have biased the study findings towards favorable outcomes. Consistency between this real-world study’s findings and the prior clinical study’s findings suggests this bias may be minimal. Unlike a clinical trial, in this real-world analysis we did not actively solicit complaints. Adverse event characterization is presented for completeness but comparison to safety data from clinical trials is limited. Second, only patients who chose to complete the device-prompted postural holds and tremor improvement ratings were included in the analysis, and a substantial number of patients and sessions were not analyzed for effectiveness due to missing or poor-quality data. Treatment effectiveness estimates may be skewed if patient measurement likelihoods were tied to satisfaction of post-therapy tremor. Third, patients were prompted to perform postural holds for measuring tremor only immediately before and after stimulation sessions, and as a result, tremor measurements in this study did not allow characterization of duration of post-stimulation treatment effect. Other clinical studies have estimated this duration to be an hour for many patients, and passively monitored at-home data may provide means to characterize this more broadly in the future [15,16]. Fourth, this study captured TAPS efficacy after 1+ years of repeated use for some patients, which extends the 90-day efficacy established in prior clinical trials [15]. TAPS became available for HCP prescription in late 2019; as the therapy continues to be available to patients for longer periods of time, future analyses that characterize multi-year safety and efficacy would be valuable. Fifth, confounding factors such as caffeine, alcohol and medications are not controlled for in real-world usage. A further limitation of the study is that wrist-based accelerometry measures the joint-interaction torques produced by the hand tremor and not the hand tremor itself. The accelerometer on-board the stimulation device measures wrist motion, and not hand or finger tremor. However, previous studies have correlated the wrist-based accelerometer measurements of tremor power with gold-standard TETRAS clinical ratings [15]. Finally, key patient-reported outcomes on activities of daily living were only assessed once and via a voluntary survey, which may be subject to recency and respondent-selection bias.

In conclusion, this real-world study reinforces and extends prior clinical trial findings on safety and durable efficacy of TAPS for tremor management in individuals with ET. Patient survey data demonstrate that patients prefer TAPS over standard of care options, including medication and surgery, and some patients who use TAPS reduce or discontinue medication. Future work to refine TAPS dosing and delivery and expand seamless motor symptom monitoring could further optimize patient and HCP therapeutic experience.
